# thanks to referees 2014

**DOI:** 10.1093/annonc/mdu521

**Published:** 2014-12-16

The Editor-in-Chief and Associate Editors of Annals of Oncology would like to extend their sincere appreciation to all those who have worked as referees for the Journal. Thank you for sharing your time, effort and professional expertise. Once again we are happy to acknowledge here that the continuing success of the Journal depends on our referees' ongoing enthusiasm and support.

The following have worked as referees for the Journal during the 12-month period October 2013 – September 2014.

Matti Aapro, *Genolier, Switzerland*
Firas Abdollah, *Detroit, USA*
Marc Abramowicz, *Brussels, Belgium*
K. Absolom, *Leeds, UK*
Jaffer A. Ajani, *Houston, USA*
Hideyuki Akaza, *Tokyo, Japan*
Munira Akhter, *Petaling Jaya, Malaysia*
Kathy Albain, *Maywood, USA*
Laurence Albiges, *Villejuif, France*
Walter Albrecht, *Mistelbach, Austria*
Heike Allgayer, *Mannheim, Germany*
Martin Almquist, *Lund, Sweden*
Tarek Tawfik Amin, *Cairo, Egypt*
Asim Amin, *Charlotte, USA*
Barbara L. Andersen, *Columbus, USA*
Masahiko Ando, *Nagoya, Japan*
Thierry André, *Paris, France*
Luca Ansaloni, *Bergamo, Italy*
Emmanuel S. Antonarakis, *Baltimore, USA*
Mickael Aoun, *Bruxelles, Belgium*
Jorge Aparicio, *Valencia, Spain*
Giuseppe Aprile, *Boston, USA*
Nadir Arber, *Tel-Aviv, Israel*
Jann Arends, *Freiburg, Germany*
Athanassios Argiris, *San Antonio, USA*
Philippe Armand, *Boston, USA*
Andrew Armstrong, *Durham, USA*
Monica Arnedos, *Villejuif, France*
Dirk Arnold, *Freiburg, Germany*
Patrick Arveux, *Dijon, France*
Carlo Aschele, *Genoa, Italy*
Melissa Assel, *New York, USA*
Riccardo Audisio, *Liverpool, UK*
Livia Silvia Augustin, *Toronto, Canada*
Dagfinn Aune, *London, UK*
Ahmad Awada, *Brussels, Belgium*
Cihan Ay, *Vienna, Austria*
Hatem A. Azim Jr, *Brussels, Belgium*
Asfar S. Azmi, *Detroit, USA*
David Azria, *Montpellier, France*
Thomas Bachelot, *Lyon, France*
Ashraf Badros, *Baltimore, USA*
Emilio Bajetta, *Monza, Italy*
Lodovico Balducci, *Tampa, USA*
Judith Balmaña, *Barcelona, Spain*
Aristotle Bamias, *Athens, Greece*
Hiroko Bando, *Tsukuba, Japan*
Malgorzata Joanna Banys, *Tuebingen, Germany*
Isobel Barnes, *Oxford, UK*
H. Bartelink, *Amsterdam, The Netherlands*
Ute K. Bartels, *Toronto, Canada*
Jennifer L. Barton, *Portland, USA*
Victor Barzey, *London, UK*
Julie K. Bassett, *Melbourne, Australia*
Matteo Bassetti, *Udine, Italy*
Tracy T. Batchelor, *Boston, USA*
Philippe Bedard, *Toronto, Canada*
Yves Beguin, *Liège, Belgium*
Yazid Belkacemi, *Creteil, France*
John Bennett, *Rochester, USA*
Jonas Bergh, *Stockholm, Sweden*
Ake Berglund, *Uppsala, Sweden*
Lothar Bergmann, *Frankfurt, Germany*
Photis Beris, *Athens, Greece*
Mogens Bernsdorf, *Copenhagen O, Denmark*
Dominik Rudolph Berthold, *Lausanne, Switzerland*
Paul Berveiller, *Paris, France*
Benjamin Besse, *Villejuif, France*
Philippe Beuzeboc, *Paris, France*
Jörg Beyer, *Zurich, Switzerland*
Giampaolo Bianchini, *Milan, Italy*
Francois Clement Bidard, *Paris, France*
Laura Biganzoli, *Prato, Italy*
Charlotte Billiet, *Leuven, Belgium*
Helgi Birgisson, *Uppsala, Sweden*
Peter Colin Black, *Vancouver, Canada*
Joan Bladé, *Barcelona, Spain*
Pierre Blanchard, *Villejuif, France*
Giovanni Blandino, *Rome, Italy*
Christian U. Blank, *Amsterdam, The Netherlands*
Harry Bleiberg, *Brussels, Belgium*
David Blum, *Trondheim, Norway*
Stefania Boccia, *Rome, Italy*
Mogens Karsbøl Boisen, *Herlev, Denmark*
Michel Bolla, *Grenoble, France*
Corrado Boni, *Reggio Emilia, Italy*
Martina Bonifazi, *Milan, Italy*
Hervé Bonnefoi, *Bordeaux, France*
Marcelo R. Bonomi, *Winston-Salem, USA*
Michael A. Bookman, *Tucson, USA*
Ivan Borbath, *Brussels, Belgium*
Josep Maria Borràs, *Hospitalet, Spain*
Gautam Borthakur, *Houston, USA*
Gerard Bos, *Maastricht, The Netherlands*
Cristina Bosetti, *Milan, Italy*
Andre Bosly, *Mont Goddine, Belgium*
Christine Bouchardy, *Geneva, Switzerland*
Béatrice Bouvard, *Angers, France*
E. Bow, *Winnipeg, Canada*
Michael Boyiadzis, *Pittsburgh, USA*
Federico Bozzetti, *Milan, Italy*
Sergio Bracarda, *Arezzo, Italy*
Raffaella Bracci, *Ancona, Italy*
Etienne Brain, *Saint Cloud, France*
Michael Paul Brown, *Adelaide, Australia*
Wolfram Brugger, *Villingen-Schwenningen, Germany*
Francois Brunotte, *Dijon, France*
Tomas Buchler, *Prague, Czech Republic*
Jeroen Buijsen, *Maastricht, The Netherlands*
Krzysztof Bujko, *Warsaw, Poland*
Ronald M. Bukowski, *Cleveland, USA*
Earle Burgess, *Charlotte, USA*
Michael David Burkitt, *Liverpool, UK*
Phyllis Butow, *Sydney, Australia*
Aman U. Buzdar, *Houston, USA*
Adele Caldarella, *Florence, Italy*
Raffaele Califano, *Manchester, UK*
Melanie Calvert, *Birmingham, UK*
Ana Paula Campanelli, *São Paulo, Brazil*
Gabriel Capella, *L'Hospitalet Barcelona, Spain*
Federico Cappuzzo, *Livorno, Italy*
Michele Caraglia, *Naples, Italy*
Chiara Carlomagno, *Naples, Italy*
William E. Carson, *Columbus, USA*
Oriol Casanovas, *Barcelona, Spain*
Stefano Cascinu, *Ancona, Italy*
Philippe Cassier, *Lyon, France*
Carlos Arturo Castaneda, *Lima, Peru*
Daniel Castellano, *Madrid, Spain*
Vincenzo Catalano, *Pesaro, Italy*
Carlo V. Catapano, *Bellinzona, Switzerland*
Laura Catena, *Monza, Italy*
Richard Cathomas, *Chur, Switzerland*
Guido Cavaletti, *Monza, Italy*
Josep Joan Centelles, *Barcelona, Spain*
Stefano Cereda, *Milan, Italy*
Maria Cerone, *London, UK*
M. Chamuleau, *Amstedsam, The Netherlands*
S. C. Chang, *Taipei, Taiwan*
Kari Chansky, *Seattle, USA*
Cecile Charles, *Villejuif, France*
Ian Chau, *Sutton, UK*
X. Chen, *New York, USA*
Ronald C. Chen, *Chapel Hill, USA*
Yi-Bin Chen, *Boston, USA*
Gautier Chene, *St Etienne, France*
B. S. Chera, *Chapel Hill, USA*
Bruce David Cheson, *Washington, USA*
Alberto Alejandro Chiappori, *Tampa, USA*
Benoist Chibaudel, *Paris, France*
Elena Gabriela Chiorean, *Indianapolis, USA*
Bartosz Chmielowski, *Los Angeles, USA*
Toni K. Choueiri, *Boston, USA*
Verslype Chris, *Leuven, Belgium*
Elisabeth Coart, *Louvain-La-Neuve, Belgium*
Veronique Cocquyt, *Ghent, Belgium*
Jan Willem W. Coebergh, *Rotterdam, The Netherlands*
Francesco Cognetti, *Rome, Italy*
Ezra E. W. Cohen, *La Jolla, USA*
Jonathon B. Cohen, *Atlanta, USA*
N. R. Cohen, *Southampton, UK*
Gabriella Cohn-Cedermark, *Stockholm, Sweden*
Bertrand Coiffier, *Pierre-Bénite, France*
Robert E. Coleman, *Sheffield, UK*
R. Coleman, *Houston, USA*
Marco Colleoni, *Milan, Italy*
PierFranco Conte, *Padua, Italy*
Romain Coriat, *Paris, France*
Paolo Corradini, *Milan, Italy*
Pippa G. Corrie, *Cambridge, UK*
Giovanni Corso, *Milan, Italy*
Javier Cortés, *Barcelona, Spain*
Alexis Benjamin Cortot, *Lille, France*
Carmen Criscitiello, *Milan, Italy*
Adam Csordas, *Innsbruck, Austria*
P. Cuijpers, *Amsterdam, The Netherlands*
Kevin J. Cullen, *Baltimore, USA*
M. P. Curado, *Ecully, France*
Jack Cuzick, *London, UK*
Sanja Dacic, *Pittsburgh, USA*
Luigino Dal Maso, *Aviano, Italy*
Francesco d'Amore, *Århus, Denmark*
Bruno Daniele, *Naples, Italy*
Daniel C. Danila, *New York, USA*
Prajnan Das, *Houston, USA*
Michael Davies, *Houston, USA*
Pradip De, *Sioux Falls, USA*
Evandro de Azambuja, *Brussels, Belgium*
Berardino De Bari, *Lausanne, Switzerland*
Filippo de Braud, *Milan, Italy*
Jacques De Greve, *Brussels, Belgium*
Filippo De Marinis, *Rome, Italy*
Sabino De Placido, *Naples, Italy*
Mark De Ridder, *Brussels, Belgium*
Eduardo De Stefani, *Montevideo, Uruguay*
Alfonso De Stefano, *Naples, Italy*
Herbert Decaluwé, *Leuven, Belgium*
Lore Decoster, *Brussels, Belgium*
Lino Del Pup, *Aviano, Italy*
Jan M. A. Delabie, *Toronto, Canada*
Pierre Delanaye, *Liège, Belgium*
Catherine Delbaldo, *Paris, France*
Julie Delyon, *Paris, France*
Ronald DeMatteo, *New York, USA*
Christiana Demetriou*, Nicosia, Cyprus*
Francesca Demichelis, *Trento, Italy*
Gary Deng, *New York, USA*
Carsten Denkert, *Berlin, Germany*
Tom C. DeRoche, *Winston-Salem, USA*
Jayesh Desai, *Melbourne, Australia*
Christine Desmedt, *Brussels, Belgium*
Frank Detterbeck, *New Haven, USA*
Eric Deutsch, *Villejuif, France*
Kapil Dhingra, *Sparta, USA*
Giuseppe Di Lorenzo, *Naples, Italy*
Massimo Di Maio, *Naples, Italy*
Mario Dicato, *Luxembourg, Luxembourg*
Paul Dickman, *Stockholm, Sweden*
Rodrigo Dienstmann, *Seattle, USA*
Michaela Dinan, *Durham, USA*
Maurizio D'Incalci, *Milan, Italy*
Anne-Marie C. Dingemans, *Maastricht, The Netherlands*
Christian Dittrich, *Vienna, Austria*
Henrik J. Ditzel, *Odense C, Denmark*
Sophie F. Doisneau-Sixou, *Toulouse, France*
Sylvie Dolbeault, *Paris, France*
Riccardo Dolcetti, *Aviano, Italy*
Julien Dômont, *Villejuif, France*
Xueyuan Dong*, Atlanta, USA*
P. Donnelly, *Nijmegen, The Netherlands*
Michael Doubek, *Brno, Czech Republic*
Tom Dowling, *Maryland, USA*
Carolyn Dresler, *Silver Springs, USA*
Martin Dreyling, *Munich, Germany*
Gypsyamber D'Souza, *Baltimore, USA*
Peter Dubsky, *Vienna, Austria*
Michel Pierre Ducreux, *Villejuif, France*
Arkadiusz Z. Dudek, *Minneapolis, USA*
Eric Duell, *Barcelona, Spain*
Reinhard Dummer, *Zurich, Switzerland*
Sarah N. Dumont, *Villejuif, France*
Ignacio Duran, *Madrid, Spain*
Francine Durocher*, Quebec, Canada*
Rafal Dziadziuszko, *Gdansk, Poland*
Wilfried Ernst Erich Eberhardt, *Essen, Germany*
Martin J. Edelman, *Baltimore, USA*
Shin Egawa, *Tokyo, Japan*
Alexander M. M. Eggermont, *Villejuif-Paris, France*
A. Eisbruch, *Ann Arbor, USA*
Nagi S. El Saghir, *Beirut, Lebanon*
Esther Elishaev, *Pittsburgh, USA*
Joerg Ellinger, *Bonn, Germany*
Cathy Eng, *Houston, USA*
Andreas Engert, *Cologne, Germany*
Meira Epplein, *Nashville, USA*
Vanessa Estall, *Melbourne, Australia*
M. S. Ewer, *Houston, USA*
Martine Extermann, *Tampa, USA*
Stefano Fagiuoli, *Bergamo, Italy*
Sandrine Faivre, *Clichy, France*
Carole Fakhry, *Baltimore, USA*
Mahdi Fallah, *Heidelberg, Germany*
Huaping Fan, *Aurora, USA*
Francoise Farace, *Villejuif, France*
Fabio Farinati, *Padua, Italy*
Stephen Farrell, *Dallas, USA*
Gianpiero Fasola, *Udine, Italy*
Matteo Fassan, *Verona, Italy*
Massimo Federico, *Modena, Italy*
Pierre Fenaux, *Paris France*
Felix Yi-Chung Feng, *Ann Arbor, USA*
Timothy Fenske, *Milwaukee, USA*
Sandra Viviana Fernandez, *Philadelphia, USA*
Gabriella Ferrandina, *Rome, Italy*
Annamaria Ferrero, *Turin, Italy*
Patrizia Ferroni, *Rome, Italy*
Maryse Fiche, *Lausanne, Switzerland*
Joan Figueras, *Girona, Spain*
Remond J. A. Fijneman, *Amsterdam, The Netherlands*
Michelangelo Fiorentino, *Boston, USA*
Rebecca Fitzgerald, *Cambridge, UK*
Aude Flechon, *Lyon, France*
Uta E. Flucke, *Nijmegen, The Netherlands*
Joakim Folkesson, *Uppsala, Sweden*
Gunnar Folprecht, *Dresden, Germany*
Arlene Forastiere, *Baltimore, USA*
Laura P. Forsythe, *Washington, USA*
Catherine Fortpied, *Brussels, Belgium*
Adam E. Frampton, *London, UK*
Wilbur Franklin, *Aurora, USA*
Stephen J. Freedland, *Durham, USA*
Alex Freeman, *London, UK*
Jonathan Friedberg, *Rochester, USA*
Hitoshi Fujiwara, *Kyoto, Japan*
Minoru Fukuchi*, Japan*
Susan Galandiuk, *Louisville, USA*
Matthew Galsky, *New York, USA*
F. Gao, *Singapore*
Francisco Javier Garcia del Muro Solans*, Barcelona, Spain*
Rocio Garcia-Carbonero, *Seville, Spain*
Elizabeth Garrett-Mayer, *Charleston, USA*
Pere Gascon, *Barcelona, Spain*
Oliver Gautschi, *Lucerne, Switzerland*
Paola Gazzaniga, *Rome, Italy*
Geraldine Gebhart, *Brussels, Belgium*
Val Gebski, *Camperdown, Australia*
Akihiko Gemma, *Tokyo, Japan*
Alessandra Gennari, *Pisa, Italy*
Suzanne George, *Boston, USA*
Vassilis Georgoulias, *Heraklion, Greece*
Marco Gerlinger, *UK*
Michele Ghielmini, *Bellinzona, Switzerland*
Rachel J. Gibson, *Adelaide, Australia*
Johannes Maria Giesinger, *Innsbruck, Austria*
F. J. Giles, *San Antonio, USA*
Silke Gillessen, *St. Gallen, Switzerland*
Antonio Giordano, *Philadelphia, USA*
Luca Giovanella, *Bellinzona, Switzerland*
Jordi Giralt, *Barcelona, Spain*
Christian Gisselbrecht, *Paris, France*
Bertram Glass, *Hamburg, Germany*
Olivier Glehen, *Pierre-Benite, France*
Bonnie Glisson, *Houston, USA*
Robert Glynne-Jones, *Northwood, UK*
Michael Gnant, *Vienna, Austria*
Daniel Gomez, *Houston, USA*
Anthony G. Goncalves, *Marseille, France*
Ajay K. Gopal, *Seattle, USA*
Margot Gosney, *Reading, UK*
Koichi Goto, *Kashiwa, Japan*
Ramaswamy Govindan, *St. Louis, USA*
Andre Goy, *Hackensack, USA*
Wilhelm Graf, *Uppsala, Sweden*
Trine Grantzau, *Denmark*
Frank Anthony Greco, *Nashville, USA*
Peter Grimison, *Sydney, Australia*
Petros Grivas, *Greece*
Harry J. M. Groen, *Groningen, The Netherlands*
Kenneth Grossman, *Salt Lake City, USA*
A. Grothey, *Rochester, USA*
Viktor Gruenwald, *Hannover, Germany*
Valentina Guarneri, *Padua, Italy*
Henk-Jan Guchelaar, *Leiden, The Netherlands*
E. Gudmundsdóttir, *Stockholm, Sweden*
Severine Guiu, *Montpellier, France*
James Gulley, *Bethesda, USA*
Ulf Gunnarsson, *Umeå, Sweden*
Marianne Guren, *Oslo, Norway*
Ralf Gutzmer, *Hannover, Germany*
Daniel Haber, *Charlestown, USA*
Thomas Matthew Habermann, *Rochester, USA*
Peyman Hadji, *Frankfurt Am Main, Germany*
Hans Hagberg, *Uppsala, Sweden*
Noah M. Hahn, *Indianapolis, USA*
Eric Hahnen, *Germany*
Susan Halabi, *Durham, USA*
Peter Hammerman, *Boston, USA*
Ji-Youn Han, *Goyang, Republic of Korea*
D. K. Hapangama, *UK*
Noboru Hara, *Niigata, Japan*
Hideyuki Harada, *Nagaizumi, Japan*
Taishi Harada, *Fukuoka, Japan*
James D. Harrison, *San Francisco, USA*
Lauren C. Harshman, *Boston, USA*
Noboru Hattori, *Hiroshima, Japan*
Axel Hauschild, *Kiel, Germany*
Jo S. Haviland, *Southampton, UK*
Ming-Liang He, *Hong Kong, Hong Kong*
S. Heggie*, UK*
Volker Heinemann, *Munich, Germany*
Viola Heinzelmann-Schwarz, *Basel, Switzerland*
Michael Henderson, *Melbourne, Australia*
Daniel Heng, *Calgary, Canada*
Christophe Hennequin, *Paris, France*
Peter Hersey, *Sydney, Australia*
Daniel L. Hertz, *Ann Arbor, USA*
Georg Hess, *Mainz, Germany*
Viviane Hess, *Basel, Switzerland*
Aram Francis Hezel, *Rochester, USA*
Toyoaki Hida, *Nagoya, Japan*
Manuel Hidalgo, *Madrid, Spain*
Catherine Hill, *Villejuif, France*
Axel Hinke, *Langenfeld, Germany*
Laurien D. C. Hoefnagel, *Utrecht, The Netherlands*
Harald J. Hoekstra, *Groningen, The Netherlands*
Otto Hoekstra, *Amsterdam, The Netherlands*
Wolf-K. Hofmann, *Mannheim, Germany*
Martin Höglund, *Uppsala, Sweden*
Peter Hohenberger, *Mannheim, Germany*
Antoine Hollebecque, *Villejuif, France*
Loes M. Hollestein, *Rotterdam, The Netherlands*
Maija Hollmén, *Zurich, Switzerland*
Blanca Homet, *Los Angeles, USA*
Krisztian Homicsko, *Lausanne, Switzerland*
Ruey-Long Hong, *Taipei, Taiwan*
Jean-Claude Horiot, *Genolier, Switzerland*
Alan Horwich, *Sutton, UK*
Steven Horwitz, *New York, USA*
Katsuyuki Hotta, *Okayama, Japan*
Chuen Hsueh, *Taoyuan Hsien, Taiwan*
Robert A. Huddart, *Sutton, UK*
Clifford Hudis, *New York, USA*
Edwin P. Hui, *Shatin, Hong Kong*
Pervin Hurmuz, *Ankara, Turkey*
Sara A. Hurvitz, *Los Angeles, USA*
Martin Hutchings, *Copenhagen, Denmark*
Chian-Yaw Hwang, *Taipei, Taiwan*
Barry Iacopetta, *Nedlands, Australia*
Shinsuke Iida, *Nagoya, Japan*
David H. Ilson, *New York, USA*
Akira Inoue, *Sendai, Japan*
E. A. Isenring, *Australia*
Fumihiro Ishida, *Matsumoto, Japan*
Farhad Islami, *Atlanta, USA*
M. G. Ison, *Chicago, USA*
Antoine Italiano, *Bordeaux, France*
Hidemi Ito, *Nagoya, Japan*
Yuri Ito, *Osaka, Japan*
S. Percy Ivy, *Bethesda, USA*
Takayuki Iwamoto, *Okayama, Japan*
Yukihide Iwamoto, *Fukuoka, Japan*
Masako Iwanaga, *Tokyo, Japan*
Renuka Iyer, *Buffalo, USA*
Koji Izutsu, *Tokyo, Japan*
Arnaud Jaccard, *Limoges, France*
William Jacot, *Montpellier, France*
Elaine S. Jaffe, *Bethesda, USA*
Patrick Jahn, *Halle (Saale), Germany*
Tae Jung Jang, *Gyongju, Republic of Korea*
Aminah Jatoi, *Rochester, USA*
Anu Jayaram, *UK*
Roxanne Jensen*, USA*
Soren Astrup Jensen, *Copenhagen, Denmark*
Yan Jiang, *Ann Arbor, USA*
Yan Jiang, *Houston, USA*
Heikki Joensuu, *Helsinki, Finland*
Markus Joerger, *St.Gallen, Switzerland*
Thomas John, *Heidelberg, Australia*
Peter W. M. Johnson, *Southampton, UK*
Melissa Johnson, *Chicago, USA*
Florence Joly, *Caen, France*
Robert Jones, *Glasgow, UK*
Anthony Joshua, *Toronto, Canada*
Seungyoun Jung, *Baltimore, USA*
Malik Juweid, *Amman, Jordan*
Tsunehisa Kaku, *Fukuoka, Japan*
M. M. Kameda-Smith, *Glasgow, Scotland, UK*
Chang Hyun Kang, *Seoul, Republic of Korea*
Gyeong Hoon Kang, *Seoul, Republic of Korea*
Nimmi S. Kapoor, *Orange, USA*
Katherine Karakoula, *Wolverhampton, UK*
R. Kawachi*, Japan*
Marina D. Kaymakcalan, *Boston, USA*
Damien Kee, *East Melbourne, Australia*
Daniel Keizman, *Kfar-Saba, Israel*
Hirotsugu Kenmotsu, *Shizuoka, Japan*
Belinda E. Kiely, *Sydney, Australia*
Merril Kies, *Houston, USA*
Young Hak Kim, *Kyoto, Japan*
Eun-Kyung Kim, *Seoul, Republic of Korea*
Tae Min Kim, *Seoul, Republic of Korea*
Hoguen Kim, *Seoul, Republic of Korea*
Scott Y. H. Kim*, USA*
Tae Won Kim, *Seoul, Republic of Korea*
Won Seog Kim, *Seoul, Republic of Korea*
Yoon Jun Kim, *Republic of Korea*
Tomohiro Kinoshita, *Nagoya, Japan*
John Kirkwood, *Pittsburgh, USA*
Shaye Kivity, *Tel Hashomer, Israel*
Alice Koechlin, *Lyon, France*
Jim Koeller, *Austin, USA*
Yasuhiro Koh, *Wakayama-Shi, Japan*
Fariba Kolahdooz, *Edmonton, Canada*
Christian Kollmannsberger, *Vancouver, Canada*
Yoshinobu Komai, *Chiba, Japan*
R. Komaki, *Houston, USA*
Scott Kopetz, *Houston, USA*
Pawel Krawczyk, *Lublin, Poland*
Nicolaus Kroger, *Hamburg, Germany*
Deborah A. Kuban, *Houston, USA*
Kaoru Kubota, *Japan*
Ragini Kudchadkar, *Tampa, USA*
Jean-Emmanuel Kurtz, *Strasbourg, France*
KyuBum Kwack, *Seongnam-si, Republic of Korea*
Carlo La Vecchia, *Milan, Italy*
Mario E. Lacouture, *New York, USA*
Angela Lamarca, *Manchester, UK*
Jeffrey Landercasper, *La Crosse, USA*
Loic Lang-Lazdunski, *London, UK*
Emilie Lanoy, *Villejuif, France*
James M. Larkin, *London, UK*
Vincent Launay-Vacher, *Paris, France*
Cecile Le Pechoux, *Villejuif, France*
Patrick Leavey, *Dallas, USA*
Frederic Lecouvet, *Brussels, Belgium*
Lee Leddy, *Charleston, USA*
Jonathan A. Ledermann, *London, UK*
Choon-Taek Lee, *Seongnam, Republic of Korea*
Celine Lefebvre, *Villejuif, France*
Natasha B. Leighl, *Toronto, Canada*
Michael F. Leitzmann, *Regensburg, Germany*
Marc Lemort, *Brussels, Belgium*
Riccardo Lencioni, *Pisa, Italy*
Daniel John Lenihan, *Nashville, USA*
John P. Leonard, *New York, USA*
Toni Lerut, *Leuven, Belgium*
T. Letilovic, *Zagreb, Croatia*
Christine Levy, *Paris, France*
Serge Leyvraz, *Lausanne, Switzerland*
Zhongxing Liao, *Houston, USA*
Stuart Marvin Lichtman, *Commack, USA*
Lisa Licitra, *Milan, Italy*
Nancy U. Lin, *Boston, USA*
Sabine S. Linn, *Amsterdam, The Netherlands*
Jakob Linseisen, *Neuherberg, Germany*
Alan Lipton, *Hershey, USA*
Jennifer Litton, *Houston, USA*
Lorenzo Livi, *Florence, Italy*
Roger Lo, *Los Angeles, USA*
Christian Loehberg, *Germany*
Patrick J. Loehrer, *Indianapolis, USA*
Sara Lonardi, *Italy*
Georgina V. Long, *Sydney, Australia*
Teri Longacre*, Stanford, USA*
Per Eystein Lonning, *Bergen, Norway*
Jochen Lorch, *Boston, USA*
Florian Lordick, *Leipzig, Germany*
Y. Loriot, *France*
Domenica Lorusso, *Milan, Italy*
Pat Lorusso, *Detroit, USA*
Izidore S. Lossos, *Miami, USA*
Fotios Loupakis, *Pisa, Italy*
Heinz Ludwig, *Vienna, Austria*
Vivian Lui, *Hong Kong, Hong Kong*
Lars Lundell, *Stockholm, Sweden*
Matthew Lunning, *Omaha, USA*
Alberto Luporini, *Milan, Italy*
Manfred P. Lutz, *Saarbrücken, Germany*
Brigette B. Y. Ma, *Shatin, Hong Kong*
Cynthia X. Ma, *St. Louis, USA*
Teresa Macarulla, *Barcelona, Spain*
Jean-Pascal Machiels, *Brussels, Belgium*
Dharanija Madhavan, *Germany*
Robert Maki, *New York, USA*
Andreas Makris, *Middlesex, UK*
Nuria Malats, *Madrid, Spain*
David G. Maloney, *Seattle, USA*
Atsushi Manabe, *Japan*
Mario Mandala, *Bergamo, Italy*
Fabrizio Marcucci, *Rome, Italy*
Maurie Markman, *Philadelphia, USA*
Miguel Martin, *Madrid, Spain*
Erika Martinelli, *Naples, Italy*
Elena Martinez Navarro, *Torrevieja, Spain*
Eva Martinez-Balibrea, *Badalona, Spain*
Jose Martin-Moreno, *Spain*
Renato Goncalves Martins, *Seattle, USA*
Anna Martling, *Stockholm, Sweden*
Celine Mascaux, *Aurora, USA*
Georg Maschmeyer, *Potsdam, Germany*
Christophe Massard, *Villejuif, France*
Christine Mateus, *Paris, France*
Itaru Matsumura, *Osaka-Sayama, Japan*
Keitaro Matsuo, *Fukuoka, Japan*
Eve Maubec, *Paris, France*
Tim Maughan, *Oxford, UK*
Joan Maurel, *Barcelona, Spain*
Dimitris Mavroudis, *Heraklion, Greece*
J. Mazieres, *France*
Tara McCannel, *Los Angeles, USA*
N. McCarthy, *Brisbane, Australia*
B. J. McCranor, *Baltimore, USA*
Howard L. McLeod, *Tampa, USA*
Ranee Mehra, *Philadelphia, USA*
Begoña Mellado, *Barcelona, Spain*
Jaime Rafael Merchan, *Miami, USA*
Melinda S. Merchant, *Bethesda, USA*
Marco Merlano, *Cuneo, Italy*
Wilma E. Mesker, *Leiden, The Netherlands*
Karen Messer, *La Jolla, USA*
Giulio Metro, *Perugia, Italy*
Paul A. Meyers, *New York, USA*
Rosalba Miceli, *Milan, Italy*
Michael Michael, *Melbourne, Australia*
Jean-Pierre Michel, *Geneva, Switzerland*
Stefan Michiels, *Villejuif, France*
Frederick Millard, *La Jolla, USA*
David Scott Miller, *Dallas, USA*
Bernard Milleron, *Paris, France*
Michael J. Millward, *Perth, Australia*
Emmanuel Mitry, *Saint-Cloud, France*
Yasushi Miyazaki, *Nagasaki, Japan*
Alden A. Moccia, *Bellinzona, Switzerland*
Dominik Paul Modest, *Munich, Germany*
Supryia G. Mohile, *Rochester, USA*
Peter Mohr, *Buxtehude, Germany*
Alexander Molassiotis, *Manchester, UK*
Miguel Angel Molina, *Barcelona, Spain*
Michele Molinari, *Halifax, Canada*
Silvio Monfardini, *Milan, Italy*
Bradley J. Monk, *Phoenix, USA*
Clara Montagut, *Barcelona, Spain*
Cristina Montomoli, *Pavia, Italy*
Malcolm J. Moore, *Canada*
Erica Moretti, *Prato, Italy*
Mitsuru Mori, *Sapporo, Japan*
William G. Morice, *Rochester, USA*
Satoshi Morita, *Yokohama, Japan*
Francoise Mornex, *Lyon-Pierre-Benite, France*
Gary Morrow, *Rochester, USA*
Alison Moskowitz, *New York, USA*
Javid Moslehi, *Boston, USA*
Adrian Muenscher, *Hamburg, Germany*
K. Müllner, *Budapest, Hungary*
Haruyasu Murakami, *Shizuoka, Japan*
Mark Murakami*, USA*
Gwen Murphy, *Bethesda, USA*
Cristina Nadal, *Spain*
Chisato Nagata, *Gifu, Japan*
Kenichi Nakamura, *Tokyo, Japan*
Takashi Nakano, *Nishinomiya, Japan*
Joo-Hyun Nam, *Seoul, Republic of Korea*
Peter Naredi, *Umea, Sweden*
Andrea Necchi, *Milan, Italy*
Elise Neol-Savina, *Brest, France*
John Neoptolemos, *Liverpool, UK*
Patrick Neven, *Leuven, Belgium*
Anthony Nichols, *Canada*
Craig Nichols, *Portland, USA*
Dorte Lisbeth Nielsen, *Herlev, Denmark*
Seiji Niho, *Kashiwa, Japan*
Yuzuru Niibe, *Chuo-ku, Japan*
Mef Nilbert, *Copenhagen, Denmark*
Saroj Niraula, *Winnipeg, Canada*
Donato Nitti, *Padua, Italy*
Naoyuki Nogami, *Matsuyama, Japan*
Josep Nomdedeu, *Barcelona, Spain*
Bernard Nordlinger, *Boulogne, France*
Peter H. Nørgaard, *Herlev, Denmark*
Nicola Normanno, *Naples, Italy*
Peter Nygren, *Uppsala, Sweden*
Mary E O'Brien, *Sutton, UK*
Norma O'Donovan, *Dublin, Ireland*
Karin Oechsle, *Hamburg, Germany*
Shuji Ogino, *Boston, USA*
Michinori Ogura, *Suzuka, Japan*
Yuichiro Ohe, *Tokyo, Japan*
Satoshi Oizumi, *Sapporo, Japan*
Morihito Okada, *Hiroshima, Japan*
Isamu Okamoto*, Japan*
Hiroaki Okamoto, *Yokohama, Japan*
Noboru Okamura, *Japan*
Yasuhiro Oki, *Houston, USA*
Yusuke Okuma, *Tokyo, Japan*
Jan Oldenburg, *Oslo, Norway*
Esther Natalie Oliva, *Reggio Calabria, Italy*
Sara Olson, *New York, USA*
Michael Ong, *Ottawa, Canada*
Nicola Orsini, *Stockholm, Sweden*
Hirotaka Osada, *Nagoya, Japan*
Piet Ost, *Ghent, Belgium*
Gyula Ostoros, *Budapest, Hungary*
Patrick A. Ott, *USA*
Sai-Hong Ignatius Ou, *Orange, USA*
Stéphane Oudard, *Paris, France*
Michael J. Overman, *Houston, USA*
Geoffrey R. Oxnard, *Boston, USA*
Simon Pacey, *Cambridge, UK*
Sun Ha Paek, *Republic of Korea*
Lars Påhlman, *Uppsala, Sweden*
P. K. Paik, *New York, USA*
Soonmyung Paik, *Pittsburgh, USA*
Channing Paller, *Baltimore, USA*
Emanuela Palmerini, *Bologna, Italy*
Richard Palmqvist, *Umeå, Sweden*
Xavier Paoletti, *Paris, France*
Helle Pappot, *Copenhagen, Denmark*
Henry Soo-Min Park, *New Haven, USA*
Keunchil Park, *Seoul, Republic of Korea*
Ben Park, *Baltimore, USA*
Ann Partridge, *Boston, USA*
Nora Pashayan, *UK*
Rodolfo Passalacqua, *Cremona, Italy*
Sapna Pradyuman Patel, *Houston, USA*
Patricia Pautier, *Villejuif, France*
Steve Pavletic, *Bethesda, USA*
Fedro A. Peccatori, *Milan, Italy*
Markus Peck-Radosavljevic, *Vienna, Austria*
Bárbara Peleteiro, *Porto, Portugal*
Nicoletta Pella, *Udine, Italy*
Frederique Penault-Llorca, *Clermont-Ferrand, France*
Nicolas Penel, *Lille, France*
Elicia Penuel, *SanFrancisco, USA*
J. Alejandro Pérez-Fidalgo, *Valencia, Spain*
Maurice Perol, *Lyon, France*
Gianfranco Angelo Pesce, *Bellinzona, Switzerland*
Milos Pesek, *Czech Republic*
Solange Peters, *Lausanne, Switzerland*
Carmel Pezaro, *Melbourne, Australia*
Georg Pfeiler, *Vienna, Austria*
David Pfister, *New York, USA*
Philip A. Philip, *Detroit, USA*
Pierpaolo Piccaluga, *Bologna, Italy*
Jean-Yves Pierga, *Paris, France*
Jean-Pierre Pignon, *Villejuif, France*
Stefano A. Pileri, *Bologna, Italy*
Dietmar Pils, *Vienna, Austria*
Marion Pineros, *Bogotá, Colombia*
Carmen Pinol Felis, *Lleida, Spain*
Robert Pirker, *Vienna, Austria*
Vlad Popovici, *Czech Republic*
Pierluigi Porcu, *Columbus, USA*
Graeme J. Poston, *Liverpool, UK*
Thomas Powles, *London, UK*
Aleix Prat, *Barcelona, Spain*
Jennifer Prescott*, USA*
Timothy Jay Price, *Woodville, Australia*
Miles Prince, *Melbourne, Australia*
Sandhya Pruthi, *Rochester, USA*
Alan Quirk*, UK*
Elisabeth Quoix, *Strasbourg, France*
Bernardo Rapoport, *Johannesburg, South Africa*
Dana Rathkopf, *New York, USA*
Stefano Rausei, *Varese, Italy*
Paolo Ravelli, *Italy*
M. Reck, *Germany*
Nicholas Reed, *Glasgow, UK*
Enrique Regidor, *Madrid, Spain*
Angelika Reiner, *Vienna, Austria*
Laurent Remontet, *Lyon, France*
Michele Reni, *Milan, Italy*
Lazzaro Repetto, *Sanremo, Italy*
Jeremy Rich, *Cleveland, USA*
Gregory J. Riely, *New York, USA*
Stephen Riggs, *Charlotte, USA*
David L. Rimm, *New Haven, USA*
Carla Ida Ripamonti, *Milan, Italy*
James Rocco, *Boston, USA*
C. Rödel, *Frankfurt, Germany*
Jordi Rodon, *Barcelona, Spain*
Sabine Rohrmann, *Zurich, Switzerland*
Fausto Roila, *Terni, Italy*
Eve Roman*, UK*
Valentina Rosato, *Milan, Italy*
Peter Graham Rose, *Cleveland, USA*
Jonathan E. Rosenberg, *New York, USA*
Antonio Rossi, *Avellino, Italy*
Marie-Claude Rousseau, *Laval, Canada*
Philippe Rousselot, *France*
Roman Rouzier, *Paris, France*
Patricia Roxburgh, *Glasgow, UK*
Kathryn J. Ruddy, *Rochester, USA*
Sarah M. Rudman, *London, UK*
Oscar M. Rueda, *Cambridge, UK*
Hope S. Rugo, *San Francisco, USA*
Simon Rule, *Plymouth, UK*
Gordon J. S. Rustin, *Northwood, UK*
Piotr Rutkowski, *Warsaw, Poland*
Everardo Delforge Saad, *Sao Paulo, Brazil*
Gunnar Sæter, *Oslo, Norway*
Junichi Sakamoto, *Nagoya, Japan*
April Salama, *Durham, USA*
Ramon Salazar, *Barcelona, Spain*
Lisa Salvatore, *Pisa, Italy*
Jesus F. San Miguel, *Pamplona, Spain*
Michiel van de Sande, *Leiden, The Netherlands*
Warren Sanger, *Omaha, USA*
Marc Sanson, *Paris, France*
Daniel Sargent, *Rochester, USA*
Andrea Sartore-Bianchi, *Milan, Italy*
Arun Satelli, *Houston, USA*
Jarupon Fah Sathirapongsasuti*, USA*
J. R. Satin, *Canada*
Kerry J. Savage, *Vancouver, Canada*
Maurizio Scaltriti, *New York, USA*
J. Schalken, *Nijmegen, The Netherlands*
C. Scheede-Bergdahl, *Montreal, Canada*
Charles Schiffer, *Detroit, USA*
Anne Schlesinger-Raab, *Munich, Germany*
Manuela Schmidinger, *Vienna, Austria*
Matthias Schmuth, *Innsbruck, Austria*
Ralph Schneider, *Bochum, Germany*
Denise Scholtens, *Chicago, USA*
Sarah-Jane Schramm, *Sydney, Australia*
F. H. Schröder, *Rotterdam, The Netherlands*
Scott Schuetze, *Ann Arbor, USA*
Rodney J. Scott, *Newcastle, Australia*
Florian Scotte, *Paris, France*
Martin Sebastian, *Frankfurt, Germany*
Marc Seifert*, Germany*
Tanguy Y. Seiwert, *Chicago, USA*
Akimasa Sekine, *Yokohama, Japan*
Ikuo Sekine, *Chiba, Japan*
Suresh Senan, *Amsterdam, The Netherlands*
Elżbieta Senkus, *Gdańsk, Poland*
D. Serban, *Bucharest, Romania*
M. Serfaty*, UK*
Violeta Serra, *Barcelona, Spain*
Cristiana Sessa, *Bellinzona, Switzerland*
Lena Sharp, *Sweden*
Qian Shi, *Rochester, USA*
Avichai Shimoni, *Tel-Hashomer, Israel*
Daniel Shin, *Los Angeles, USA*
Hai-Rim Shin, *Manila, Philippines*
Nicole Shonka, *Omaha, USA*
Andrei R Shustov, *Seattle, USA*
M. Simunovic, *Canada*
Susanne Singer, *Mainz, Germany*
Lillian Siu, *Toronto, Canada*
Nicole Skoetz, *Cologne, Germany*
S. F. Slovin, *New York, USA*
Egbert F. Smit, *Amsterdam, The Netherlands*
Tenbroeck Smith, *Atlanta, USA*
Alberto Sobrero, *Genoa, Italy*
Benjamin Solomon, *Melbourne, Australia*
Stephen Sonis, *Boston, USA*
Amir Sonnenblick, *Jerusalem, Israel*
Guru Sonpavde, *Birmingham, USA*
Halfdan Sorbye, *Bergen, Norway*
Pierre Soubeyran, *Bordeaux, France*
L. Soucek, *Barcelona, Spain*
Joseph A. Sparano, *Bronx, USA*
Alan Spatz, *Montreal, Canada*
Lena Specht, *Denmark*
Paul Sperduto, *Waconia, USA*
Silvia Stacchiotti, *Milan, Italy*
Walter M. Stadler, *Chicago, USA*
Elisabeth Stahle, *Sweden*
Giorgio Stassi, *Palermo, Italy*
Ewout W. Steyerberg, *Rotterdam, The Netherlands*
Stephan Stilgenbauer, *Ulm, Germany*
Richard Stone, *Boston, USA*
Florian Strasser, *St. Gallen, Switzerland*
Thomas Stricker, *Nashville, USA*
Jonathan Strosberg, *Tampa, USA*
Loreta Strumylaite, *Kaunas, Lithuania*
Roger Stupp, *Zurich, Switzerland*
Jan Styczynski, *Bydgoszcz, Poland*
Chunxia Su, *Shanghai, China*
Naoko Sueoka-Aragane, *Japan*
Kenichi Sugihara, *Tokyo, Japan*
Elizabeth Suh-Burgmann, *Walnut Creek, USA*
Eric Sun, *Stanford, USA*
Qian Sun, *USA*
Jong-Mu Sun, *Seoul, Republic of Korea*
Antonella Surbone, *New York, USA*
Stephanie Sutherland, *St Albans, UK*
Ritsuro Suzuki, *Nagoya, Japan*
Christopher Sweeney, *Boston, USA*
Matthew Sydes, *London, UK*
James Symanowski, *Charlotte, USA*
William F. Symmans, *Houston, USA*
John Syrios, *Piraeus, Greece*
Laszlo Tabar, *Falun, Sweden*
Giovanni Tafuri, *Rome, Italy*
Pierosandro Tagliaferri, *Italy*
Emanuela Taioli, *Manhasset, USA*
Masayuki Takeda, *Osaka, Japan*
Kengo Takeuchi, *Tokyo, Japan*
Nagio Takigawa, *Okayama, Japan*
Yuichi Takiguchi, *Chiba, Japan*
Eng-Huat Tan, *Singapore, Singapore*
Torgrim Tandstad, *Trondheim, Norway*
Fuminori Taniguchi, *Yonago, Japan*
Alessandra Tedeschi, *Milan, Italy*
Sabine Tejpar, *Leuven, Belgium*
Leon W. M. M. Terstappen, *Enschede, The Netherlands*
Christine A. Theodore, *Suresnes, France*
Catherine Thieblemont, *Paris, France*
Antoine Thiery-Vuillemin, *Besançon, France*
David Thomas, *Australia*
Sumitra Thongprasert, *Chiangmai, Thailand*
Fredericus B. J. M. Thunnissen, *Amsterdam, USA*
Wayne D. Tilley, *Adelaide, Australia*
Ingeborg Tinhofer, *Berlin, Germany*
Kensei Tobinai, *Tokyo, Japan*
Yuji Toiyama, *Tsu, Japan*
Sara M. Tolaney, *Boston, USA*
Shuta Tomida, *Osaka, Japan*
Naoto Tomita, *Yokohama, Japan*
Patricia Tonin, *Montreal, Canada*
Suzanne L. Topalian, *Baltimore, USA*
Valter Torri, *Milan, Italy*
David Tougeron, *Poitiers, France*
Maud Toulmonde, *Bordeaux, France*
Jennifer Treleaven, *Sutton, UK*
Pierre Triozzi, *Cleveland,USA*
S. Tsai, *Milwaukee, USA*
Ming-Sound Tsao, *Toronto, Canada*
Lambros Tselikas, *Villejuif, France*
S. M. Tu, *Houston, USA*
Samra Turajlic, *London, UK*
Clare Turnbull, *UK*
Ignasi Tusquets, *Barcelona, Spain*
Shelley S. Tworoger, *USA*
Yoshiaki Uyama, *Chiyodaku, Japan*
Ulka Vaishampayan, *Detroit, USA*
Miriam Valentini, *Santa Maria Imbaro, Italy*
Vincenzo Valentini, *Rome, Italy*
Juan Valle, *Manchester, UK*
Eric Van Cutsem, *Leuven, Belgium*
Lonneke van de Poll-Franse, *Eindhoven, The Netherlands*
Cornelis J. H. Van de Velde, *Leiden, The Netherlands*
Winette Van der Graaf, *Nijmegen, The Netherlands*
Felice Neline van Erning, *Eindhoven, The Netherlands*
Mieke Van Hemelrijck, *London, UK*
C. M. L. van Herpen, *Nijmegen, The Netherlands*
J. H. Van Krieken, *Nijmegen, The Netherlands*
C. van Scheppingen, *The Netherlands*
Zsuzsanna Varga, *Switzerland*
Enrico Vasile, *Pisa, Italy*
Lars Vatten, *Trondheim, Norway*
Nele Vermaete, *Leuven, Belgium*
Kataja Vesa, *Kuopio, Finland*
Giuseppe Viale, *Milan, Italy*
Eduardo Vilar, *Houston, USA*
Neus Villamor, *Barcelona, Spain*
Anthony Michael Villani, *Adelaide, Australia*
Mark Vincent, *London, Canada*
Anne Vincent-Salomon, *Paris, France*
Otto Visser, *Utrecht, The Netherlands*
Umberto Vitolo, *Turin, Italy*
Margaret von Mehren, *Philadelphia, USA*
Gunter von Minckwitz, *Neu-Isenburg, Germany*
Adri C. Voogd, *Eindhoven, The Netherlands*
Julie Vose, *Omaha, USA*
Te Vuong, *Montreal, Canada*
Kylie Vuong, *Camperdown, Australia*
Lars Wagner, *Lexington, USA*
Heather A. Wakelee, *Stanford, USA*
M. G. Wallis, *UK*
Diethelm Wallwiener, *Tuebingen, Germany*
Jun Wan, *Shenzhen, China*
Lu Wang, *New York, USA*
Zuomin Wang, *Beijing, China*
Qiming Wang, *Zhengzhou, China*
Shun-ichi Watanabe, *Tokyo, Japan*
Jeffrey S. Weber, *Tampa, USA*
Thomas Weber, *Halle, Germany*
Janine Wechsler, *Villejuif, France*
Ulrich Wedding, *Jena, Germany*
Joseph Wee, *Singapore*
Howard West, *Seattle, USA*
Nick West, *Leeds, UK*
Paul Wheatley-Price, *Ottawa, Canada*
Arne Wibe, *Trondheim, Norway*
Thomas Wiegel, *Ulm, Germany*
Hans Wildiers, *Leuven, Belgium*
Christopher Willett, *Durham, USA*
C. Winter, *Krefeld, Germany*
Jane Winter, *Chicago, USA*
Thomas E. Witzig, *Rochester, USA*
Jedd D. Wolchok, *New York, USA*
Ido Wolf, *Israel*
Jürgen Wolf, *Cologne, Germany*
Adrian Worrall, *UK*
Agnieszka Wozniak, *Leuven, Belgium*
Xifeng Wu, *Houston, USA*
Hongping Xia, *Singapore, Singapore*
Wei Fen Xie, *Shanghai, China*
Yuri Yamaguchi, *Japan*
Kazuhito Yamamoto, *Nagoya, Japan*
Nobuyuki Yamamoto, *Shizuoka, Japan*
Ryuya Yamanaka, *Kyoto, Japan*
Takeharu Yamanaka, *Yokohama, Japan*
Chih Hsin Yang, *Taipei, China*
Hushan Yang, *Philadelphia, USA*
Timothy Anthony Yap, *London, UK*
Yasushi Yatabe, *Nagoya, Japan*
I-Tien Yeh, *San Antonio, USA*
Sai-Ching Yeung, *Houston, USA*
Gregory S. Yochum, *USA*
Junji Yoshida, *Japan*
Takaki Yoshikawa, *Yokohama, Japan*
Naruo Yoshimura, *Osaka, Japan*
Ichiro Yoshino, *Chiba, Japan*
Danny Ross Youlden, *Brisbane, Australia*
Joanne P. Young, *Australia*
Jun Yu, *Hong Kong, Hong Kong*
Helena Alexandra Yu, *New York, USA*
John Raymond Zalcberg, *Melbourne Victoria, Australia*
K. Zaman, *Lausanne, Switzerland*
Alberto Zaniboni, *Brescia, Italy*
Dimitrios Zardavas, *Brussels, Belgium*
M. P. Zeegers, *Utrecht, The Netherlands*
Fang Fang Zhang, *Boston, USA*
Xuehong Zhang, *Boston, USA*
Caicun Zhou, *Shanghai, China*
Andrew X. Zhu, *Boston, USA*
Xiao-Ping Zou, *Nanjing, China*
Emanuele Zucca, *Bellinzona, Switzerland*

With apologies for possible errors and omissions.

